# Interrelationship of rotavirus infection and Creatine Kinase-MB isoenzyme levels in children hospitalized with acute gastroenteritis in Guangzhou, China, 2012–2015

**DOI:** 10.1038/s41598-017-07636-4

**Published:** 2017-08-09

**Authors:** Jianbin Zheng, Haiqing Zheng, Ramit Kumar Gupta, Huixian Li, Hui Shi, Liyan Pan, Sitang Gong, Huiying Liang

**Affiliations:** 1 Pediatric Intensive Care Unit, Guangzhou Women and Children’s Medical Center, Guangzhou Medical University, Guangzhou, China; 2 Institute of Pediatrics, Guangzhou Women and Children’s Medical Center, Guangzhou Medical University, Guangzhou, China; 3 Cardiac Intensive Care Unit, Heart Center, Guangzhou Women and Children’s Medical Center, Guangzhou Medical University, Guangzhou, China; 4 Department of Gastroenterology, Guangzhou Women and Children’s Medical Center, Guangzhou Medical University, Guangzhou, China

## Abstract

Elevated levels of Creatine Kinase-MB (CK-MB) Isoenzyme are a common phenomenon among rotavirus (RV) diarrhea. However, few studies have addressed this issue using large sample size. In current study, 1,118 children (age <5 years) hospitalized with diarrhea in Guangzhou Women and Children’s Medical Center from 2012 to 2015 were finally included. Changing pattern of CK-MB and its relationship with RV-infection were analyzed and characterized. Multivariate linear regression models showed that RV-positive cases had a 28% rise in CK-MB compared to RV-negative cases (OR = 1.28, 95% CI: 1.15 to 1.41, P < 0.01) after controlling for age, gender, season of admission, and weight. The pattern of change showed that CK-MB level of RV-positive group started to rise immediately at the 1^st^ day of diarrhea, reached the peak on days 2 to 4, declined during 4–9 days, and then reached a relatively stable level when compared to the RV-negative group. Mediation analyses showed that indirect effect of RV infection on the increase of CK-MB via Vesikari score was significant (β = 8.01, P < 0.01), but direct effect was not (β = 9.96, P = 0.12). Thus, elevated CK-MB value is a common finding in RV-infection and completely mediated by the severity of diarrhea. CK-MB monitoring may help to identify children with more severe viral infection.

## Introduction

Diarrhea leads to high morbidity and mortality rates in children, especially in developing countries^1^. Rotavirus (RV) is the most common cause of severe and dehydrating diarrheal disease among children aged <5 years worldwide^2^. As of April 2016, according to WHO estimates, globally 215,000 children aged <5 years die each year from vaccine-preventable rotavirus infections, and the vast majority of these children live in low-income countries^2^. In the developing countries such as China, RV infection caused approximately 47.8% of diarrhea hospitalizations among children^3^, and 53,559 children died during 2003–2012^4^. However, only 22.5% of them have received at least 1 dose of the Lanzhou lamb rotavirus (LLR) vaccine in Guangzhou^5^, which is much lower than vaccine coverage in the USA (78%)^6^.

Previous studies suggested that RV may cause some extra intestinal injuries including liver damage^7^, nephritis^8^, central nervous system complications^9^ (e.g, meningoencephalitis)^10^, exanthema^11^, myocarditis^12^, and even death^2, 12, 13^. Creatine Kinase-MB Isoenzyme (CK-MB), one form of the myocardial fraction of creatinine phosphokinase, was used extensively as an indication for myocardial damage. An elevation in CK-MB is a significant predictor of adverse heart events and was found to be correlated with myocardial infarct size^14, 15^. Recent studies suggested that RV infection may cause an increase in CK-MB Isoenzyme. For example, Shi *et al*. demonstrated that elevated CK-MB were found among 55% of the RV infected Chinese children aged 2–48 months^16^, which was subsequently confirmed by Li *et al*.^17^. Similar studies had been also conducted in Japan^18–20^ and Korea^21^. A clinical study of Tatsumi *et al*.^18^. showed that children with RV-gastroenteritis (n = 41, 1.4–6.9 years) had a significantly higher CK-MB/CK ratio (0.61 vs. 0.17) than healthy subjects (n = 55, 1.3–5.8 years). Significant difference of patients’ CK activities was also observed between RV antigen positive children (n = 14, 60.0 ± 20.6 U/I) and control children (n = 105, 7.1 ± 2.9 U/I) in the studies of Hoshino *et al*.^19, 20^.

Obviously, current evidence for the potential relationship between RV infection and CK-MB (or CK) has been mainly based on small sample size. Furthermore, only univariate analyses were considered in previous studies, which couldn’t eliminate the confounding effects from potential confounding variables. The determination of whether there is such an association has potential importance for managing RV infection, for preventing of adverse events, and for understanding mechanisms of severe complications caused by RV. Thus, a more comprehensive study with larger sample size and proper design was performed to (1) assess the association between RV infection and the level of CK-MB; (2) to explore the dynamic changes of CK-MB after RV infection; and (3) test this type of association: direct or indirect.

## Results

**Figure 1:**
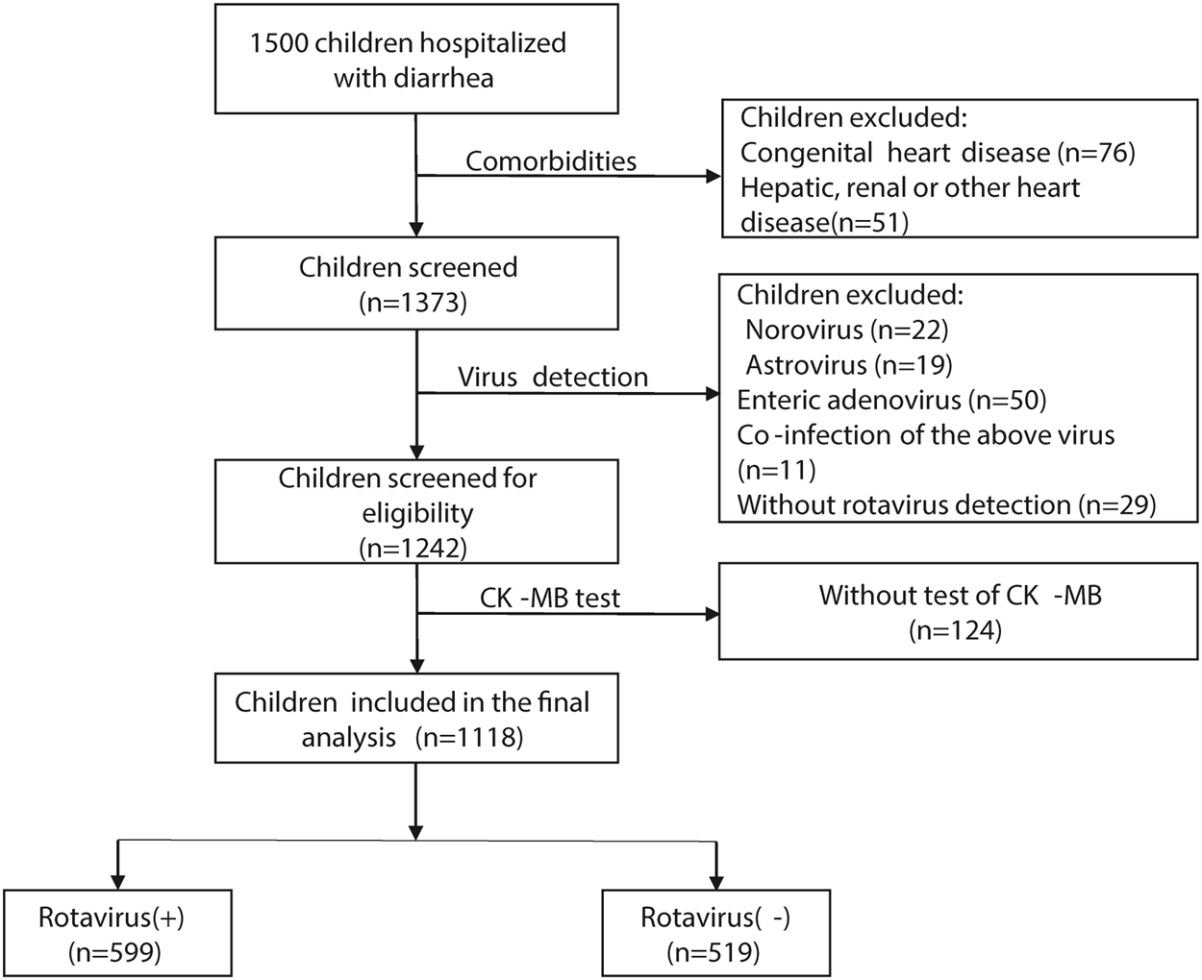
Study sample selection.

A total of 1,500 children were finally enrolled, of whom 1,118 were included in the final analyses. Three hundred and eighty-two children were not included due to the following reasons: congenital heart disease, hepatic, renal or other severe disease which might cause elevated serum CK-MB (n = 127); other viral infections or co-infections (n = 131); no information on CK-MB (n = 124) (Fig. 1).

### Participant characteristics

**Figure 2:**
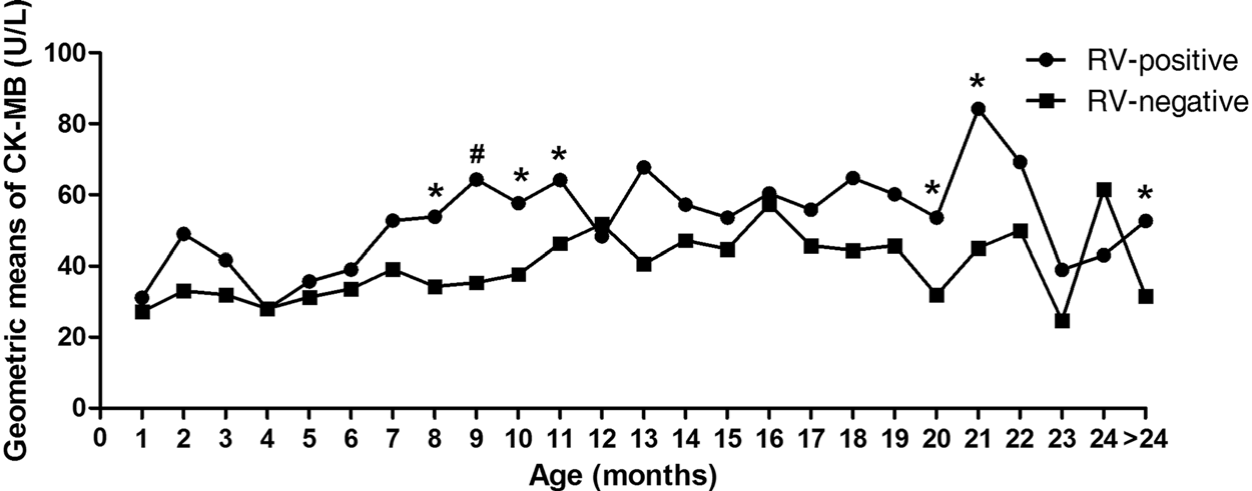
Changes of geometric means of CK-MB titers in RV-positive and RV-negative children according to age by months. CK-MB values in RV-negative children in every age group were as references. ^“*”^P < 0.05, ^“#”^P < 0.01. RV, rotavirus; CK-MB, Creatine Kinase-MB.

Characteristics of patients are summarized overall and by RV status in Table 1. Among the 1,118 subjects included in the analysis, there were 780 (69.8%) males and 599 (53.6%) RV-positive children, with a mean age of 14.31 months (SD = 10.47 months). Table 1 shows that the RV-positive children were significantly older and thus had higher body weights than the RV-negative children. Gender is equally distributed. According to the seasons in the Northern Hemisphere, winter had much higher RV-infection incidence than three other seasons. Exploratory analysis showed that both CK-MB and CK were significantly higher among RV-positive group than that of RV-negative group. Subsequently, detailed analyses were carried out for every age group in the CK-MB (Fig. 2) and CK levels (Supplementary Figure S1). As shown in Figs 2 and S1, elevated CK-MB and CK occur in almost all age groups.

**Table 1:** Characteristics of rotavirus-positive and rotavirus-negative.

Patient characteristics	Total	Rotavirus-positive	Rotavirus-negative	Χ^2^/t/z	P value
Gender					
Male	780 (69.8)	411 (52.7)	369 (47.3)	0.81	0.37
Female	338 (30.2)	188 (55.6)	150 (44.4)		
Season of admission					
Spring	120 (10.7)	61 (50.8)	59 (49.2)	122.11	<0.01
Summer	183 (16.4)	40 (21.9)	143 (78.1)		
Autumn	357 (31.9)	180 (50.4)	177 (49.6)		
Winter	458 (41.0)	318 (69.4)	140 (30.6)		
Age(months)	14.31 ± 10.47	16.61 ± 10.93	11.65 ± 9.23	8.13	<0.01^‡^
Weight(Kg)	9.20 ± 3.03	9.93 ± 2.86	8.22 ± 2.98	8.41	<0.01^‡^
CK-MB(U/L)	42 (27–74.25)	55 (30–87)	35 (24–54)	8.64	<0.01^#^
CK(U/L)	98 (64–157)	108 (75–167.25)	83 (52–142)	6.27	<0.01^#^

Data are presented as n (%) and mean ± standard deviation (SD) and median (interquartile range, IQR).

^‡^ *P-*value are shown for between-groups comparisons of values using Student t-test.

^#^ *P-*value are shown for between-groups comparisons of values using Mann Whitney U test.

CK-MB, Creatine Kinase-MB; CK, Creatine Kinase.

These results were also confirmed in multivariate analyses. As shown in Supplementary Tables S1 and S2, even after controlling for age, gender, season of admission and weight, RV-positive children still suffered a 28% rise in CK-MB (OR = 1.28, 95% CI: 1.15 to 1.41, P < 0.01) and 15% rise in CK (OR = 1.15, 95% CI: 1.01 to 1.32, P = 0.04) compared to RV-negative children.

### Dynamic changes of levels of CK-MB and CK after the onset of diarrhea

**Figure 3:**
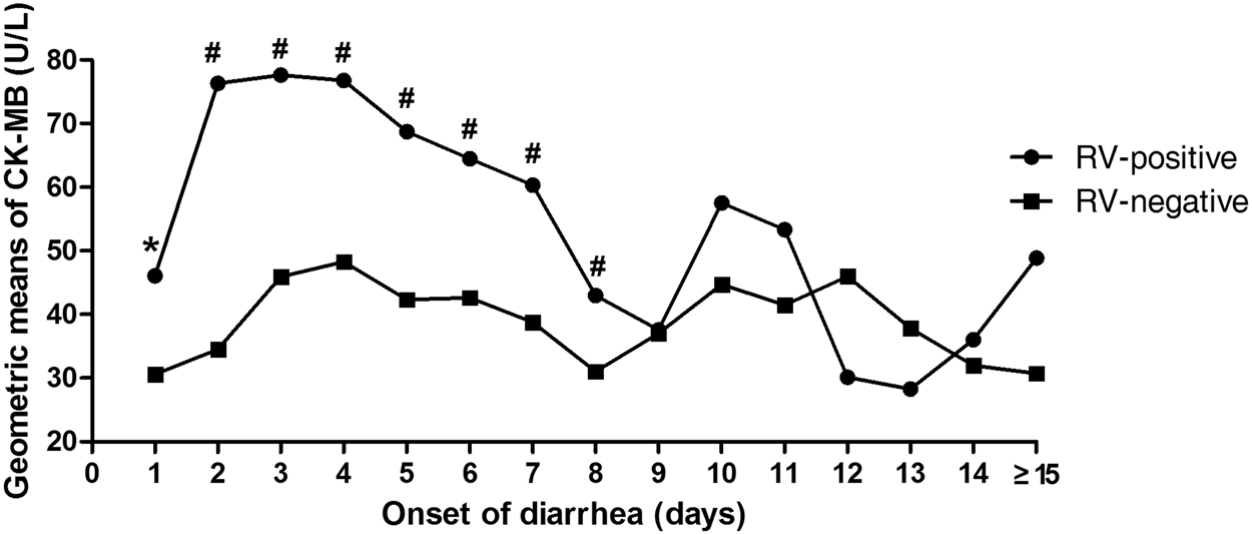
Changing patterns of geometric means of CK-MB titers in RV-positive and RV-negative children according to days after onset of diarrhea. CK-MB values in RV-negative children in every day after onset of diarrhea were as references. ^“*”^P < 0.05, ^“#”^P < 0.01. RV, rotavirus; CK-MB, Creatine Kinase-MB.

As demonstrated in Fig. 3, the level of CK-MB of patients with RV-infection was compared with patients who didn’t have RV-infection. We found that geometric means (GMs) of CK-MB increased dramatically at the first day of onset of diarrhea in the RV-infected children. Peak CK-MB values were reached on days 2 to 4, and then declined over the next 4 days to relatively stable levels. On day 1 the GMs were 46.06 U/L and 30.56 U/L in those children with and without RV-infection, respectively (P = 0.048). There were significant differences on days 1–8 between RV-positive and RV-negative children (71.05 U/L vs 38.23 U/L, P < 0.01), while no significant differences were found on days 9–14 (41.76 U/L vs 36.59 U/L, P = 0.181). Similar phenomenon were also recorded in the change of CK (Supplementary Figure S2).

### Mediating role of disease severity in the association between RV infection and CK-MB or CK level

**Figure 4:**
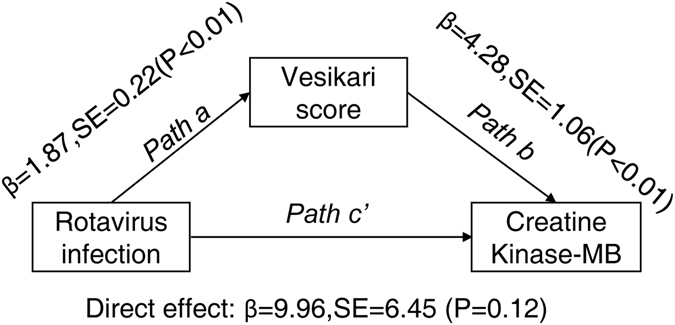
Mediation analysis of RV infection and the level of CK-MB.

Figure 4 illustrates the mediation effect of the association between RV-infection and elevated CK-MB or CK. The severity of diarrhea (Vesikari score) was a significant mediator of the association between RV-infection and elevated CK-MB. After controlling for Vesikari score, the association between the RV-infection and elevated CK-MB was no longer significant (β = 9.96, SE = 6.45, bootstrapped bias-corrected 95% CI: −2.71 to 22.63, P = 0.12), but the presence of RV-infection was significantly associated with higher Vesikari score (β = 1.87, SE = 0.22, P < 0.01), which was in turn associated with higher CK-BM (β = 4.28, SE = 1.06, P < 0.01). The indirect coefficient was significant (β = 8.01, SE = 3.48, bootstrapped bias-corrected 95% CI: 2.49 to 15.91, P < 0.01), which means that RV infection was associated with approximately 8.01 U/L higher CK-MB values as mediated by diarrhea severity. However, neither direct effect (β = 26.97, bootstrapped bias-corrected 95% CI: −92.51 to 167.63, P = 0.79) nor indirect effect (β = 37.47, bootstrapped bias-corrected 95% CI: −7.62 to 109.80, P = 0.21) of RV infection on the increase of CK via diarrhea severity were significantly (Supplementary Figure S3).

## Discussion

Most of earlier studies showed RV infection was associated with elevated CK-MB^16–18, 21^ or CK^19, 20^. However, there were several major limitations of previous studies, for example uncertainty of inclusion or exclusion criteria, no efficient comparative groups, no multivariate model to adjust for the potential confounding factors, and small sample size. Due to these limitations and based on strict inclusion and exclusion criteria, multivariate analysis, and large sample size, we assessed comprehensively the association between RV-infection and CK-MB for the first time. Results showed that rotavirus infection was associated with a 28% elevation of CK-MB (OR = 1.28, 95% CI: 1.15 to 1.41). Marginally significant association between RV-infection and elevated CK was also observed (OR = 1.15, 95% CI: 1.01 to 1.32). This may be because that CK-MB is a more sensitive marker than CK for cardiac function damage^22^.

Regrettably, as described in the introduction section, studies focusing on the association between RV infection and level of CK-MB or CK are mainly limited in East Asian countries and regions. This may be related to the different medical behavior patterns between the East and the West. For example, in China, irrespectively of what disease the patients suffer from, “extensive” blood tests will be carried out which are relevant for the functions of the heart, liver, kidney, and other organs. In Western countries, a patient admitting with diarrhea disease only receives a “precision and specific” test related to the digestive system. Therefore, there are more clinical and biochemical data in Eastern countries to support searches for correlations. The mechanism by which the CK-MB and CK levels are raised by RV infection is not known. Elevation of CK-MB or cardiac troponin (cTn) had been observed among dengue virus and respiratory syncytial virus infection patients^23, 24^. Furthermore, myocarditis and dilated cardiomyopathy had been observed among hepatitis C virus patients^25, 26^. Both our and previous results suggested that virus infection may injury myocardium through certain mechanisms. Obviously, to some extents, these remind us to pay close attention to the change of an indicator of myocardial injury when facing children with diarrhea.

It is of great clinical significance to understand the changing pattern of CK-MB after RV infection. In this study, we used geometric means of CK-MB after the onset of diarrhea to explore patterns of change in CK-MB levels. CK-MB increased immediately at the first day of onset of diarrhea in the RV-infected children and peak CK-MB values were reached on days 2 to 4. The CK-MB peak after 1 day is earlier than the findings of Shi and his colleagues^16^, who found that the peak CK-MB value was reached on days 6 to 9. This inconsistency may be related to the difference of study population, (1) patients in the Shi *et al*. study were much younger than those of current study (6.7 months vs. 14.31 months), and (2) including many patients with myocardial injury. However, both studies suggest that there is a window period on the detection of abnormal cardiac function among children with diarrhea. Thus, for common children with RV diarrhea, we should take monitoring and preventive measures timely within this period of three days. In addition, to assess the interrelationship between RV infection and the level of CK-MB, this study explored the potential type of this association: direct or indirect? Results showed that severity of diarrhea completely mediated the effect of RV infection on CK-MB change. Some previous published papers may help to explain the mediating role of the severity of diarrhea in the association between RV-infection and elevated CK-MB. For example, Li *et al*. discovered a positive association between the serum myocardial enzyme and the severity of disease among children with RV-infected diarrhea^27^. Two other studies also found that both the level of CK-MB and the proportion of elevated CK-MB of the severe disease group were higher than those in the mild disease group^28, 29^. However, Huang *et al*. found no association between severity of disease and degree of myocardial injury among 116 RV-infected children^30^. These inconsistencies could be attributed to differences in the severity of diarrhea. Because the criteria adopted by Huang *et al*. could not comprehensively evaluate the severity of the disease compared to the total Vasikari score commonly used. There is a possible mechanism by which the severity of diarrhea may mediate the effect of RV-infection on the elevation of CK-MB: (1) rotavirus infection causes severe diarrhea, (2) severe diarrhea results in a loss of electrolytes and dehydration^31^, and (3) electrolyte imbalances, particularly the lack of potassium, makes cardiac muscle irritable and leads to the increasing of CK-MB^32^.

However, this study has some limitations which have to be pointed out. First, given the nature of retrospective observational study design, many meaningful parameters were unavailable. For example, cTn I has been proved to be a very sensitive tool to detect minor myocardial injury^14, 15, 33^, but cTn measurements were not available for the present study. A further deficit of present study is the lack of data on viral antigen or nucleic acids in blood. In that case, we can analyze the relationship between CK-MB level and the load of RV in circulation. Second, we hypothesized that CK-MB can be used to identify children at high risk for cardiac function injury from diarrhea hospitalizations. But regrettably, for children admitted with diarrhea, we lacked more detailed data of cardiac symptoms or signs to confirm the clinical practice. Third, the pattern of CK-MB change after RV-infection was fitted mainly based on the data from different patients at different time points. Thus, a prospective study should be conducted with a series of consecutive plasma CK-MB value to acquire a more accurate change pattern. Fourth, future studies to establish the mechanisms are warranted to ascertain the elevation of CK-MB after RV infection.

## Conclusion

An elevated CK-MB value is a common finding in children with rotavirus diarrhea, with a peak during days 2–4 of acute illness. The association between RV-infection and elevated CK-MB was significantly mediated by the severity of the diarrhea. These findings may provide a novel insight into the mechanism by which RV-infection induced CK-MB elevation and in the therapeutic implications of diarrhea-related complications.

## Methods

### Study population

Acute diarrhea was defined as three or more loose, or looser-than-normal stools during a 24-hour period and significant changes in the stool exterior, including watery textures, mucous, or thin paste but excluded the presence of pus or blood^34^. Children admitting between Jan 1, 2012 and Dec 31, 2015 to the Department of Gastroenterology of Guangzhou Women and Children’s Medical Center (GWCMC) and meeting the following inclusion criteria were enrolled: (1) hospitalized with acute diarrhea, (2) younger than 5 years of age. Exclusion criteria comprised children with congenital heart disease, hepatic, renal or other severe disease which might cause elevated serum CK-MB levels. This study was approved by the Medical Ethics Committee of Guangzhou Women and Children’s Medical Center. And the methods in the study were conducted in accordance with the guidelines of the Declaration of Helsinki. Written informed consent was obtained from all the guardians of the participants.

### Data and stool collection

Stool specimens were collected from each patient in a sterile stool container. After collection, the samples were held at room temperature for no longer than 3 hours before freezing and were kept frozen until testing.

Trained study staff reviewed medical records to extract the relevant data, then recorded them on a standardized case report form. Information collection included the child’s demographics, anthropometric measurements, date of admission, diarrheal treatment and outcome, the number of days hospitalized, and laboratory tests, especially the laboratory findings of CK-MB and CK isoenzymes.

### Assessment of severity of diarrhea

The enrolled child’s clinical information of frequency and duration of diarrhea and vomiting, extent of fever, and dehydration before admission were derived from standardized interviews with the attending clinician, and from medical record abstraction. The severity of diarrhea was evaluated using the 20-point Vesikari scoring system based on the longest duration and peak frequency of diarrhea and vomiting, extent of fever, degree of dehydration, and treatment provided^35^ by using the data at admission. A higher score (up to 20) indicates more severe diarrhea.

### Laboratory virus testing

Once a week, stool specimens were tested for the presence of rotavirus by the immune colloidal gold technique using a commercial kit-Rotascreen^®^ Dipstick (Beijing Wantai Biological Pharmacy Enterprise Co. Ltd., Beijing, China) according to the manufacturer’s instructions. Norovirus, astrovirus, and adenovirus were detected by Polymerase Chain Reaction (PCR) using the Quantitative Polymerase Chain Reaction Detecting System ABI7500 (Applied Biosystems, America) as described previously^36^. To rule out the impacts of other viruses, non-rotavirus infection or co-infection were excluded from the final analysis.

### Statistical analysis

Categorical variables were expressed as the percentage and compared by Chi-square test. Continuous variables were reported as mean (standard deviation, SD) or median (interquartile range, IQR) and compared using the Student’s t-test or Mann-Whitney test, where appropriate. CK-MB and CK levels were natural log-transformed due to skewness. Multivariate linear regression models of CK-MB levels were used to estimate odds ratios (OR) and 95% confidence intervals (95% CI) for influencing factors. For children with multiple CK-MB and CK isoenzymes examinations, only the first one were included in the analyses.

Geometric mean (GM) and geometric standard deviation (GSD) of CK-MB and CK titers for each age group and days after onset of diarrhea were calculated. GSD represents the antilog of SD of the arithmetic mean of the log enzyme titers, indicating that the variance of the GM represents a range of GM multiplied or divided by the GSD. And when compared data and how they changed with age and days after the onset of diarrhea, we used a line graph.

The mediating effect of the severity of the diarrhea on the relationship between the RV-infection and elevated CK-MB was tested using the three-step regression described by Baron and Kenny^37^ & Cerin and MacKinnon^38^. First, testing the direct relationship between RV infection and CK-MB; second, testing the effect of RV infection on Vesikari score; and third, testing the effect of Vesikari score on CK-MB after controlling for RV infection. Mediation is demonstrated when the main independent variable (i.e., RV-infection) is significantly associated with the main dependent variable (i.e., CK-MB level); the independent variable (i.e., RV-infection) is significantly associated with the mediator variable (i.e., Vesikari score); and the mediator variable (i.e., Vesikari score) is significantly associated with the dependent variable (i.e., CK-MB level) when the independent variable (i.e., RV-infection) is controlled. Bootstrapped bias corrected 95% CIs of the direct and indirect effects were computed with 5000 bootstrapped samples.

All of the P values were two-sided and statistical significance was assessed at P = 0.05. The analysis was conducted using R Version 3.3.2 and GraphPad Prism 5.0.

## Electronic supplementary material

Supplementary materials
