# Supplementary material for: Interrelationship of rotavirus infection and Creatine Kinase-MB isoenzyme levels in children hospitalized with acute gastroenteritis in Guangzhou, China, 2012–2015
Source: Sci Rep. 2017 Aug 9;7:7674. doi: 10.1038/s41598-017-07636-4 (PMC5550499; doi:10.1038/s41598-017-07636-4)
Supplement: Supplementary file 1 — Supplementary materials [file 41598_2017_7636_MOESM1_ESM.pdf]

Title page

Title: Interrelationship of rotavirus infection and creatine kinase-MB isoenzyme levels in children hospitalized with acute gastroenteritis in Guangzhou, China, 2012-2015

Authors: Jianbin Zheng[1,#], Haiqing Zheng[2,#], Ramit Kumar Gupta[3], Huixian Li[2], Hui Shi[2], Liyan Pan[2], Sitang Gong[4,*] & Huiying Liang[2,*]

Affiliations:

[1] Pediatric Intensive Care Unit, Guangzhou Women and Children's Medical Center, Guangzhou Medical University, Guangzhou, China;

[2] Institute of Pediatrics, Guangzhou Women and Children's Medical Center, Guangzhou Medical University, Guangzhou, China;

[3] Cardiac Intensive Care Unit, Heart Center, Guangzhou Women and Children's Medical Center, Guangzhou Medical University, Guangzhou, China;

[4] Department of Gastroenterology, Guangzhou Women and Children's Medical Center, Guangzhou Medical University, Guangzhou, China;

Correspondence and requests for materials should be addressed to H-Y. L (lianghuiying@hotmail.com) or S. G (sitanggong@126.com).

Address: No.9 Jinsui Road, Zhujiang Newtown, Tianhe District, Guangzhou 510623, China.

J.Z and H.Z contributed equally to this manuscript.

H-Y.L and S.G contributed equally to this manuscript.

**Variables**	**β**	**SE**	**OR**	**95%CI**	**P value**
Age(months)	-0.01	0.01	0.99	0.98-0.99	<0.01
Sex(female vs. male)	0.19	0.52	1.21	0.74-0.91	<0.01
Season of admission (vs. winter)					
Spring	-0.17	0.08	0.84	0.72-0.98	0.03
Summer	-0.36	0.07	0.69	0.61-0.81	<0.01
Autumn	-0.14	0.06	0.87	0.78-0.98	0.02
RV infection (positive vs. negative)	0.25	0.05	1.28	1.15-1.41	<0.01
Weight (Kg)	0.06	0.01	1.06	1.03-1.09	<0.01
Constant	3.56	0.10	35.16	28.93-43.03	<0.01

Supplementary Table S1. Relationship between RV-positive and RV-negative children and logarithm of CK-MB according to the multivariate linear regression analysis.

SE, standard error; CI, confidence interval; OR, odds ratio; RV, rotavirus; CK-MB, Creatine Kinase-MB.

Variables	β	SE	OR	95%CI	P value
Age(months)	-0.01	0.01	0.99	0.98-1.00	0.04
Sex(female vs. male)	-0.11	0.07	0.89	0.78-1.02	0.10
Season of admission (vs. winter)					
Spring	-0.03	0.11	0.97	0.79-1.20	0.81
Summer	-0.25	0.01	0.78	0.64-0.94	0.01
Autumn	-0.09	0.08	0.91	0.79-1.06	0.24
RV infection (positive vs. negative)	0.14	0.07	1.15	1.01-1.32	0.04
Weight (Kg)	0.07	0.02	1.07	1.03-1.11	<0.01
Constant	4.32	0.14	75.19	57.39-98.19	4.32

Supplementary Table S2. Relationship between RV-positive and RV-negative children and logarithm of CK according to the multivariate linear regression analysis.

SE, standard error; CI, confidence interval; OR, odds ratio; RV, rotavirus; CK, Creatine Kinase.

	RV-positive			RV-negative		
**Age (months)**	**n**	**GM**	**GSD**	**n**	**GM**	**GSD**
1	7	31.09	1.84	22	27.20	1.78
2	16	48.97	2.57	47	33.01	1.92
3	12	41.72	1.91	27	31.99	2.98
4	11	27.99	1.80	28	28.02	1.47
5	23	35.67	1.52	35	31.28	1.82
6	21	39.01	2.39	18	33.59	2.02
7	24	52.72	1.82	25	39.16	1.86
8	21	53.79*	2.37	24	34.32	1.77
9	23	64.33#	1.88	27	35.39	1.88
10	35	57.63*	1.97	19	37.71	1.72
11	25	64.14*	1.77	22	46.33	1.66
12	34	48.32	1.75	32	51.71	1.94
13	23	67.69	2.45	19	40.61	2.09
14	31	57.17	1.86	25	47.13	2.43
15	25	53.57	2.07	22	44.74	1.78
16	32	60.38	1.86	17	57.28	1.95
17	32	55.81	2.05	13	45.69	2.07
18	18	64.70	1.93	9	44.34	1.80
19	12	60.09	1.93	7	45.69	1.95
20	17	53.57*	1.95	9	31.91	1.57
21	17	84.18*	1.96	7	45.02	1.77
22	7	69.19	2.34	7	49.99	1.64
23	15	38.94	2.38	6	24.72	1.76
24	9	43.01	1.61	3	61.43	1.79
>24	109	52.63#	2.11	49	31.50	1.90

Supplementary Table S3. Geometric means of CK-MB titers in RV-infection and RV-negative children according to age by months. "*", P<0.05, "#", P<0.01. GM,geometric mean; GSD, geometric standard deviation; RV, rotavirus; CK-MB, Creatine Kinase-MB.

	RV-positive			RV-negative		
**Age (months)**	**n**	**GM**	**GSD**	**n**	**GM**	**GSD**
1	7	130.82	1.87	22	76.15	2.19
2	16	103.35	2.86	47	66.79	2.17
3	12	102.17	2.36	27	80.87	2.72
4	11	103.54	1.79	28	109.53	3.02
5	23	114.45	2.11	35	91.94	2.11
6	21	153.35	3.86	18	95.94	3.33
7	24	112.58	1.78	25	94.33	2.22
8	21	116.66	3.49	24	92.49	2.69
9	23	122.48	1.58	27	85.09	2.39
10	35	119.78	2.64	19	86.13	2.06
11	25	115.05	1.66	22	93.61	1.74
12	34	98.89	1.59	32	111.05	3.48
13	23	144.44	3.79	19	86.98	1.93
14	31	136.66	2.94	25	90.32	3.27
15	25	98.83	1.54	22	99.68	2.44
16	32	109.54	1.98	17	146.04	3.82
17	32	156.96	2.16	13	125.55	4.87
18	18	103.08	1.64	9	103.69	2.18
19	12	141.08	2.01	7	106.67	5.56
20	17	134.10	2.67	9	80.17	3.12
21	17	193.38	2.86	7	116.71	1.97
22	7	113.14	2.21	7	148.41	2.06
23	15	115.17	2.93	6	72.67	2.46
24	9	163.06	2.17	3	175.38	2.09
>24	109	121.87	2.49	49	105.64	3.01

Supplementary Table S4. Geometric means of CK titers in RV-positive and RV-negative children according to age by months. GM,geometric mean; GSD, geometric standard deviation; RV,rotavirus; CK, Creatine Kinase.

	RV-positive			RV-negative		
**Onset of diarrhea (days)**	**n**	**GM**	**GSD**	**n**	**GM**	**GSD**
1	51	46.06*	2.39	42	30.56	1.80
2	79	76.32#	1.89	80	34.52	1.89
3	82	77.62#	1.61	72	45.93	2.09
4	61	76.79#	1.75	60	48.29	2.84
5	73	68.72#	2.11	68	42.33	1.97
6	50	64.49#	1.89	56	42.64	2.42
7	62	60.33#	2.43	70	38.76	1.74
8	51	42.95#	2.07	44	31.02	1.92
9	30	37.57	1.62	26	37.02	1.93
10	10	57.52	2.29	32	44.71	1.89
11	22	53.35	2.64	19	41.45	1.82
12	25	30.11	1.59	30	45.97	2.09
13	39	28.29	1.77	21	37.85	2.23
14	20	36.04	2.13	19	31.99	1.43
≥15	49	48.88#	2.12	68	30.74	1.80

Supplementary Table S5. Geometric means of CK-MB titers in RV-positive and RV-negative children according to days after onset of diarrhea. "*", P<0.05, "#", P<0.01. GM,geometric mean; GSD, geometric standard deviation, RV,rotavirus, CK-MB, Creatine Kinase-MB.

	RV-positive			RV-negative		
**Onset of diarrhea (days)**	**n**	**GM**	**GSD**	**n**	**GM**	**GSD**
1	51	87.60	2.07	42	92.18	2.19
2	79	120.22	1.74	80	105.20	2.65
3	82	124.25	1.76	72	108.63	2.83
4	61	119.63	1.76	60	104.70	4.55
5	73	116.85#	1.69	68	88.42	2.14
6	50	108.24	1.81	56	90.74	5.03
7	62	83.55	1.97	70	69.09	1.81
8	51	84.25	1.64	44	86.13	1.93
9	30	69.33	1.57	26	81.38	2.53
10	10	76.54*	2.48	32	103.40	3.45
11	22	78.44*	1.79	19	107.80	3.47
12	25	120.32	2.01	30	101.97	2.28
13	39	60.81	1.66	21	87.51	4.14
14	20	84.49	1.94	19	82.56	2.62
≥15	49	84.25	1.83	68	79.12	2.64

Supplementary Table S6. Geometric means of CK titers in RV-positive and RV-negative children according to days after onset of diarrhea. "*",P<0.05, "#", P<0.01. GM,geometric mean; GSD, geometric standard deviation; RV,rotavirus; CK,Creatine Kinase.

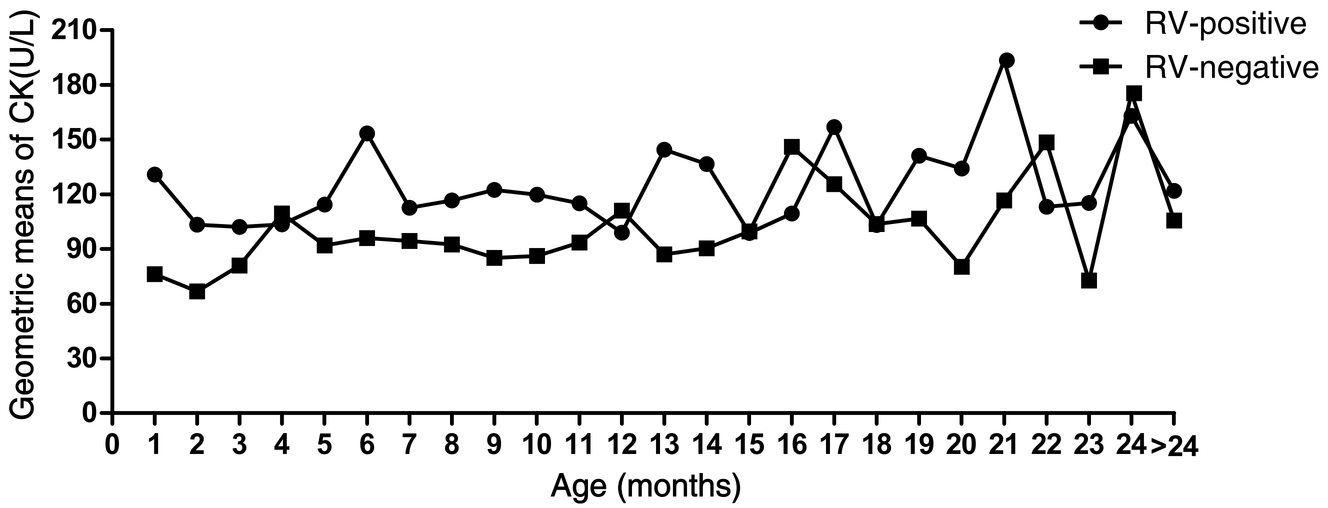

Supplementary Figure S1.Changes of geometric means of CK titers in RV-positive and RV-negative children according to age by months. RV,rotavirus; CK,Creatine Kinase.

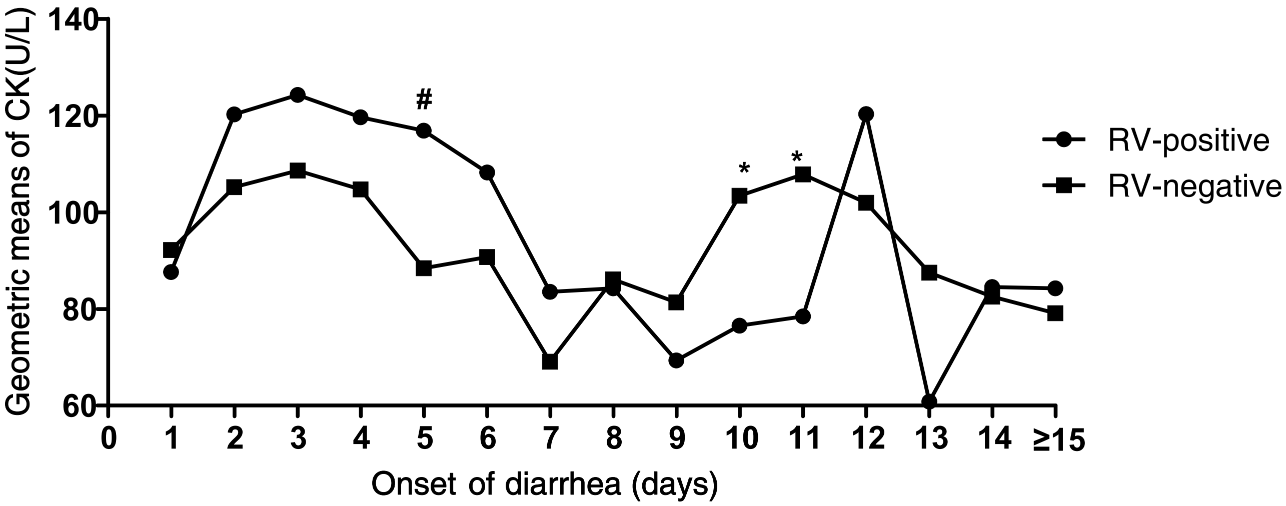

Supplementary Figure S2. Changing patterns of geometric mean of CK titers in RV-positive and RV-negative children according to days after onset of diarrhea."*",P<0.05,"#",P<0.01.
RV,rotavirus; CK,Creatine Kinase.

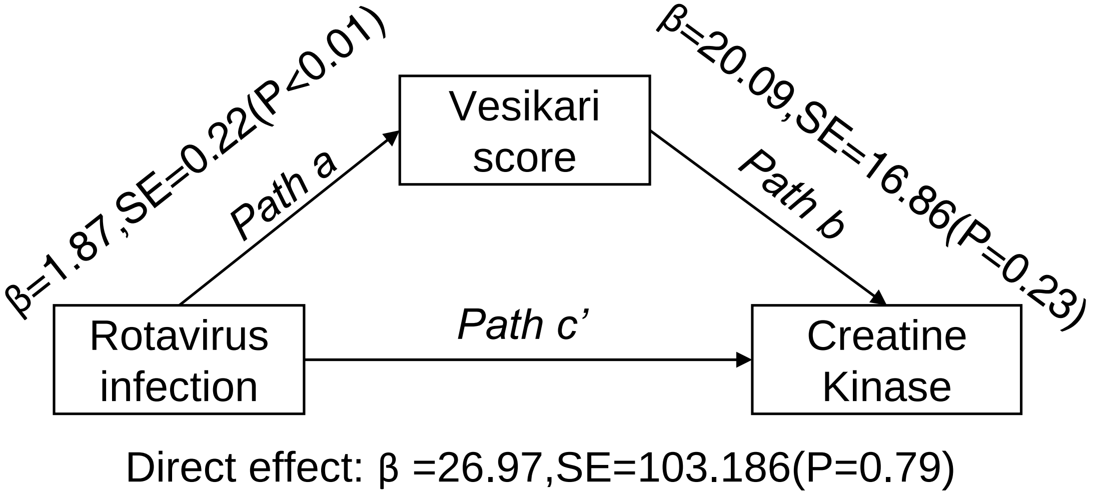

Supplementary Figure S3. Mediation analysis of rotavirus infection and the level of Creatine Kinase.
